# Modeling the evolution of disability acceptance after hypertensive intracerebral hemorrhage: a latent growth analysis

**DOI:** 10.3389/fneur.2026.1817015

**Published:** 2026-04-30

**Authors:** Zuo Lian Zhang, Xi Liu, Song Bin Huang, Meng Huang, Guo Wen Zhang, Man Li Wu, Jia Zhang

**Affiliations:** 1 Heyuan People's Hospital, Heyuan, China; 2 Heyuan Hospital of Traditional Chinese Medicine, Heyuan, China

**Keywords:** disability acceptance, hypertensive intracerebral hemorrhage, latent growth model, longitudinal study, psychological adaptation, self-efficacy

## Abstract

**Background:**

Psychological adaptation to acquired disability, conceptualized as disability acceptance, is crucial for recovery after hypertensive intracerebral hemorrhage (HICH). However, the longitudinal course of disability acceptance remains unclear.

**Objective:**

This study aimed to model the developmental trajectory of disability acceptance over six months post-discharge in HICH patients and to examine the effects of self-efficacy (time-varying) and age (time-invariant) on this trajectory.

**Methods:**

A prospective longitudinal study was conducted with 114 HICH patients. Disability acceptance and self-efficacy were assessed at discharge (T0), 1 month (T1), 3 months (T2), and 6 months (T3) post-discharge. Unconditional and conditional latent growth models (LGMs) were fitted to the data to identify the optimal growth form and to test the predictive effects of covariates.

**Results:**

The optimal trajectory was nonlinear, showing initial growth then stabilization. Self-efficacy showed a strong, time-specific positive association with concurrent disability acceptance (β: 0.522-0.562, *p* < 0.001). Age positively predicted the rate of change (slope: β= 0.309, *p* < 0.01) but not the initial level.

**Conclusion:**

Disability acceptance in HICH evolves dynamically early on. Self-efficacy consistently supports acceptance, whereas age is associated with the speed of improvement. Early interventions targeting self-efficacy could optimize psychological recovery.

## 1 Introduction

Hypertensive intracerebral hemorrhage (HICH) is a severe cerebrovascular event that frequently results in significant physical disability and profound psychological challenges for survivors (1). Beyond neurological recovery, a patient's psychological adaptation to acquired disability—a process conceptualized as disability acceptance—is a critical determinant of long-term quality of life, rehabilitation engagement, and overall functional outcome (2). Disability acceptance entails a cognitive and emotional restructuring through which individuals integrate functional limitations into a positive self-concept, thereby enabling the pursuit of meaningful life goals despite impairments (3, 4).

While cross-sectional studies have identified various factors associated with acceptance levels (5), the dynamic and evolving nature of this psychological construct after stroke necessitates a longitudinal perspective (6). Understanding its trajectory, specifically how acceptance changes over time, is essential for identifying critical windows for intervention and for discerning whether adaptation follows a linear recovery path, a decelerating curve, or reaches a stable plateau. Furthermore, delineating the factors that influence this trajectory is crucial for developing targeted support strategies. Self-efficacy, defined as the belief in one's capability to manage specific demands inherent to chronic disease, is theorized to be a potent and time-varying influence. It may exert proximal effects on acceptance at different stages of recovery (7). Conversely, demographic factors such as age may serve as time-invariant covariates, potentially shaping the overall pattern, including both the initial level and the rate of change, of the acceptance trajectory (8).

Despite its clinical importance, research that explicitly models the longitudinal course of disability acceptance in HICH populations remains scarce. Latent Growth Modeling (LGM) offers a powerful analytical framework to address this gap, as it allows for the estimation of both intra-individual change patterns and inter-individual differences in these patterns (9). Therefore, this prospective longitudinal study aimed to: (1) identify the optimal unconditional growth trajectory of disability acceptance in HICH patients over the first 6 months post-discharge; and (2) examine the predictive effects of time-varying self-efficacy and time-invariant age on this identified trajectory.

## 2 Methods

### 2.1 Participants and procedure

A prospective longitudinal study was conducted. Patients diagnosed with primary HICH were consecutively recruited from the Department of Neurosurgery at Heyuan People's Hospital, a tertiary Grade A hospital, between August 2023 and August 2025. The study protocol was approved by the Ethics Committee of Heyuan People's Hospital (Approval No: YXYJLL-2022S58) and was registered at the Chinese Clinical Trial Registry (Registration No: ChiCTR2300071778). All participants provided written informed consent prior to enrollment. The study was conducted in accordance with the Declaration of Helsinki.

The diagnosis of HICH was established according to the Chinese Guidelines for the Diagnosis and Treatment of Intracerebral Hemorrhage (2019) (10), based on the following criteria: (1) acute onset; (2) presence of focal neurological deficit symptoms; (3) evidence of hyperdense lesions on cranial CT scans indicating hemorrhage; and (4) exclusion of other non-vascular causes.

Patient selection strictly adhered to the following criteria. Inclusion criteria were: (1) age ≥18 years; (2) a history of hypertension or a new diagnosis of hypertension confirmed during the index hospitalization; (3) sufficient verbal or non-verbal communication abilities without significant cognitive impairment; and (4) provision of informed consent to participate. Exclusion criteria included: (1) a pre-existing history of mental illness, severe cognitive dysfunction, or major comorbid diseases affecting vital organs (e.g., heart, kidney, liver); (2) severe aphasia or dysarthria hindering reliable communication even with assistance; and (3) the presence of physical disabilities prior to the hemorrhagic stroke.

Assessments were conducted at four time points: at discharge (T0/baseline) and at 1, 3, and 6 months post-discharge (T1–T3). Follow-up data were collected through outpatient visits or structured telephone interviews by trained research assistants. To minimize measurement bias, all assessors underwent standardized training and followed uniform protocols. Selection bias was reduced by consecutive enrollment.

All patients received acute care and nursing according to the Chinese Guidelines for the Diagnosis and Treatment of Intracerebral Hemorrhage (2019). A total of 125 eligible patients were initially enrolled. During the 6-month follow-up, 11 patients (8.8%) had no follow-up data at any of the three post-discharge time points (1, 3, and 6 months) due to loss to follow-up (*n* = 8) or withdrawal of consent (*n* = 3), leaving 114 patients who completed all four assessments as the final analytic sample. Only participants with complete data at all time points were included. Simulation studies indicate that a sample size of 100–150 is adequate for latent growth models of moderate complexity (11). Our post-hoc Monte Carlo power analysis (12) confirmed that *N* = 114 provides sufficient statistical power for all key parameters.

### 2.2 Measures

Demographic and clinical characteristics, including age, gender, education level, and admission National Institutes of Health Stroke Scale (NIHSS) score (13), were collected using a standardized case report form designed by the research team.

Acceptance of Disability Scale (ADS): The Chinese version of the Acceptance of Disability Scale (ADS), originally developed by Linkowski (3) and later translated and validated by Chen et al. (14), was used to assess patients' cognitive and emotional adjustment to their disability. This version contains 32 items across four dimensions. The total score ranges from 32 to 128, with higher scores indicating greater acceptance.

Self-Efficacy for Managing Chronic Disease 6-Item Scale (SECD6): The SECD6, developed by Lorig et al. (15), is a validated instrument designed to assess patients' confidence in managing the multifaceted demands associated with chronic illness. Respondents indicate their level of confidence using a 10-point visual analog scale (VAS), anchored at 1 (“not at all confident”) and 10 (“completely confident”). Item scores are averaged to yield a total score, with higher values reflecting greater self-efficacy.

### 2.3 Data analysis

Descriptive statistics and bivariate correlations were computed using SPSS 23.0. The primary analysis involved Latent Growth Modeling (LGM) conducted with Mplus 8.3.

Based on the well-documented time course of stroke recovery, in which most functional gains occur within the first 3 months followed by a plateau or only minimal improvement thereafter (16), we hypothesized a growth-then-plateau pattern for disability acceptance. To test this hypothesis and identify the optimal growth form, we specified and compared four unconditional LGMs by applying different fixed time codes to the slope factor loadings: linear models with codes (0,1,2,3) and (0,1,3,6), as well as two nonlinear models with codes (0,1,3,4) and (0,1,2,2). The latter two represent alternative shapes of decelerating growth. Model fit was evaluated using multiple indices: a non-significant chi-square test (χ^2^), Comparative Fit Index (CFI) and Tucker-Lewis Index (TLI) >0.95, Root Mean Square Error of Approximation (RMSEA) < 0.08, and Standardized Root Mean Square Residual (SRMR) < 0.08. Lower values on the Akaike Information Criterion (AIC), Bayesian Information Criterion (BIC), and sample-size adjusted BIC (aBIC) indicated better fit and parsimony.

After selecting the best-fitting unconditional model as the baseline, we examined the effects of candidate covariates. For time-varying covariates, we specified a model where self-efficacy scores at each wave (T0-T3) were regressed on the concurrent ADS score. For time-invariant covariates, we tested a set of candidate variables (age, and baseline NIHSS) separately by regressing them on the latent intercept and slope factors. Covariates with significant effects (*p* < 0.05) in these separate analyses were then included together in the final full model, alongside the time-varying self-efficacy effects. The robust maximum likelihood (MLR) estimator was used. Model comparisons relied on the fit indices and the relative reduction in AIC/BIC values. Standardized regression coefficients (β) are reported. The significance level was set at *p* < 0.05 (two-tailed).

## 3 Results

### 3.1 Participant characteristics

All 114 participants completed the full 6-month follow-up period. Descriptive statistics are presented in Table 1. The mean age was 57 years (SD = 11.57). The majority were male (69.3%) and had an education level of elementary school or below (45.6%). Baseline NIHSS scores ranged from 1 to 19, indicating that our sample covered mild to moderate-severe HICH patients, which is typical for this population excluding those with extreme deficits (NIHSS >20) or severe cognitive / communication impairments. Attrition analysis (Supplementary Table S1) showed no significant baseline differences between completers (*n* = 114) and non-completers (*n* = 11, 8.8%) on any variable (all *p* > 0.05), supporting random missingness and unbiased complete-case analysis.

**Table 1:** Demographic and clinical characteristic of patients with hypertensive cerebral hemorrhage.

Characteristic	*n*	%	M	SD
Age (years)			57.00	11.57
Gender				
Male	79	69.30		
Female	35	30.70		
Education level				
Below elementary	52	45.62		
Junior high school	42	36.84		
Senior high school	13	11.40		
College or university school	7	6.14		
NIHSS				
Mild (0~15 score)	31	27.20		
Moderate (16~30 score)	59	51.75		
Severe (31~45 score)	24	21.05		
Personality types				
Introvert	41	35.97		
Neutral	56	49.12		
Extrovert	17	14.91		

### 3.2 Descriptive statistics and correlations

Cronbach's α for the ADS ranged from 0.85 to 0.91 across the four time points, while for the SECD6 it ranged from 0.88 to 0.93, indicating good to excellent internal consistency. Means, standard deviations, and intercorrelations for the study variables across four time points are presented in Table 2. Disability acceptance (ADS) scores showed a consistent increase from discharge to the 6-month follow-up, while self-efficacy (SECD6) scores also exhibited a gradual upward trend.

**Table 2:** Descriptive statistics and intercorrelations among study variables.

Variable	M	SD	ADS/T0	ADS/T1	ADS/T2	ADS/T3	SECD6/T0	SECD6/T1	SECD6/T2	SECD6/T3
ADS/T0	69.16	15.604	1.00							
ADS/T1	71.22	15.723	0.432^**^	1.00						
ADS/T2	75.31	16.400	0.314^**^	0.698^**^	1.00					
ADS/T3	77.75	19.251	0.271^**^	0.654^**^	0.804^**^	1.00				
SECD6/T0	31.25	7.846	0.300^**^	0.474^**^	0.501^**^	0.417^**^	1.00			
SECD6/T1	32.19	8.165	0.107	0.502^**^	0.525^**^	0.458^**^	0.808^**^	1.00		
SECD6/T2	34.43	8.058	0.141	0.479^**^	0.615^**^	0.516^**^	0.694^**^	0.802^**^	1.00	
SECD6/T3	35.40	8.533	0.167	0.506^**^	0.629^**^	0.557^**^	0.634^**^	0.771^**^	0.959^**^	1.00

Alpha coefficients are reported on the diagonal. ADS = The Acceptance of Disability Scale. SECD6 = Self-efficacy for Managing Chronic Disease 6-Item Scale. T0 = Time 0 (At discharge); T1 = Time 1 (1 month); T2 = Time 2 (3 months); T3 = Time 3 (6 months).

^*^p < 0.05,

^**^p < 0.01.

The correlation analysis revealed a clear pattern of relationships. All pairwise correlations among ADS scores across time points were positive and significant (*r* = 0.271 to 0.804, all *p* < 0.01), as were the correlations among SECD6 scores across time points (*r* = 0.634 to 0.959, all *p* < 0.01). Most importantly, with the exception of the non-significant correlations between self-efficacy at any time point (T0-T3) and the baseline level of disability acceptance (ADS at T0), all other correlations between self-efficacy and concurrent or subsequent disability acceptance scores were positive and significant (*r* = 0.300 to 0.629, all *p* < 0.01). This pattern preliminarily suggests that self-efficacy is more strongly associated with the concurrent and evolving state of disability acceptance than with its initial starting point, supporting its treatment as a time-varying covariate in the subsequent growth models.

### 3.3 Unconditional latent growth models

**Figure 1:**
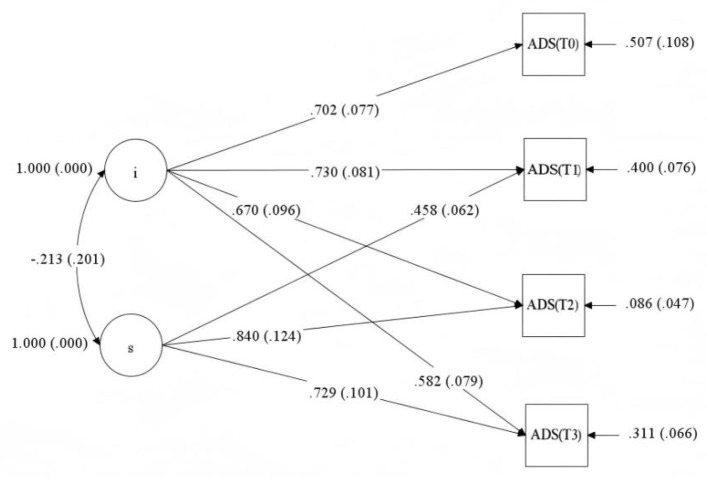
Unconditional latent growth model of acceptance of disability with standardized parameter estimates.

Fit indices for the four competing unconditional LGMs are presented in Table 3. The model specifying time codes of 0, 1, 2, 2 for the slope factor demonstrated a good fit to the data ($\chi_{\left(5\right)}^{2}$ = 7.490, *p* = 0.187; CFI = 0.985; TLI = 0.982; RMSEA = 0.066; SRMR = 0.073) and had the lowest AIC, BIC, aBIC value. This model, specified with fixed time codes of 0, 1, 2, and 2 for the slope factor loadings (as depicted in Figure 1), was tested to represent a hypothesized trajectory where disability acceptance increases from T0 to T2 and then plateaus between T2 and T3. The significant mean of the slope factor (Estimate = 3.539, *p* < 0.001) confirmed a positive overall growth trend during the active growth phase (T0 to T2). The variances of both the intercept and slope factors were significant (*p* < 0.01), indicating significant individual differences in the initial level and the rate of change in acceptance. Figure 1 presents the standardized parameter estimates for this unconditional model, including the non-significant negative correlation between the intercept and slope factors (*r* = −0.213, *p* = 0.290), which indicated no reliable evidence for a systematic relationship between initial acceptance and its subsequent rate of change in this sample.

**Table 3:** Statistical fit for proposed models in terms of latent growth curve model analysis for disability acceptance.

Model	x2 (df)	*P*-Value	AIC	BIC	Sample-adjusted BIC	RMESA	SRMR	TLI	CFI
Unconditional model (time codes: 0,1,3,6)	13.015 (5)	0.0232	3,665.487	3,690.113	3,661.667	0.119	0.158	0.941	0.951
Unconditional model (time codes: 0,1,3,4)	7.392 (5)	0.1931	3,662.226	3,686.852	3,658.406	0.065	0.137	0.982	0.985
Unconditional model (time codes: 0,1,2,3)	5.108 (5)	0.4028	3,661.894	3,686.520	3,658.074	0.014	0.108	0.999	0.999
**Unconditionalmodel (time codes: 0,1,2,2)**	**7.490 (5)**	**0.1867**	**3,660.046**	**3,684.671**	**3,656.225**	**0.066**	**0.073**	**0.982**	**0.985**
Time-varying covariate model	23.767 (17)	0.1259	3,592.121	3,627.691	3,586.603	0.059	0.064	0.968	0.975
Time-invariant covariate model	7.931 (7)	0.3387	3,655.84	3,685.938	3,651.171	0.034	0.060	0.993	0.995
**Full model (mixed covariates)**	**23.562 (19)**	**0.2135**	**3,589.453**	**3,630.496**	**3,583.086**	**0.046**	**0.057**	**0.978**	**0.984**

RMSEA = Root Mean Square Error of Approximation; SRMR = Standardized Root Mean Square Residual; TLI = Tucker–Lewis Index; CFI = Comparative Fit Index; AIC = Akaike Information Criterion; BIC = Bayesian Information Criterion. The model with time codes 0, 1, 2, 2 was selected as the baseline model due to optimal fit. Bold values indicate the models selected as the best-fitting models (the unconditional baseline model with time codes 0,1,2,2 and the full mixed covariates model).

### 3.4 Conditional latent growth models

Fit indices for the conditional models are shown in the lower part of Table 3. The full model, incorporating both time-varying self-efficacy and time-invariant age, showed excellent fit ($\chi_{\left(19\right)}^{2}$ = 23.562, *p* = 0.214; CFI = 0.984; TLI = 0.978; RMSEA = 0.046; SRMR = 0.057) and the lowest AIC, representing the most optimal and parsimonious account of the data.

**Figure 2:**
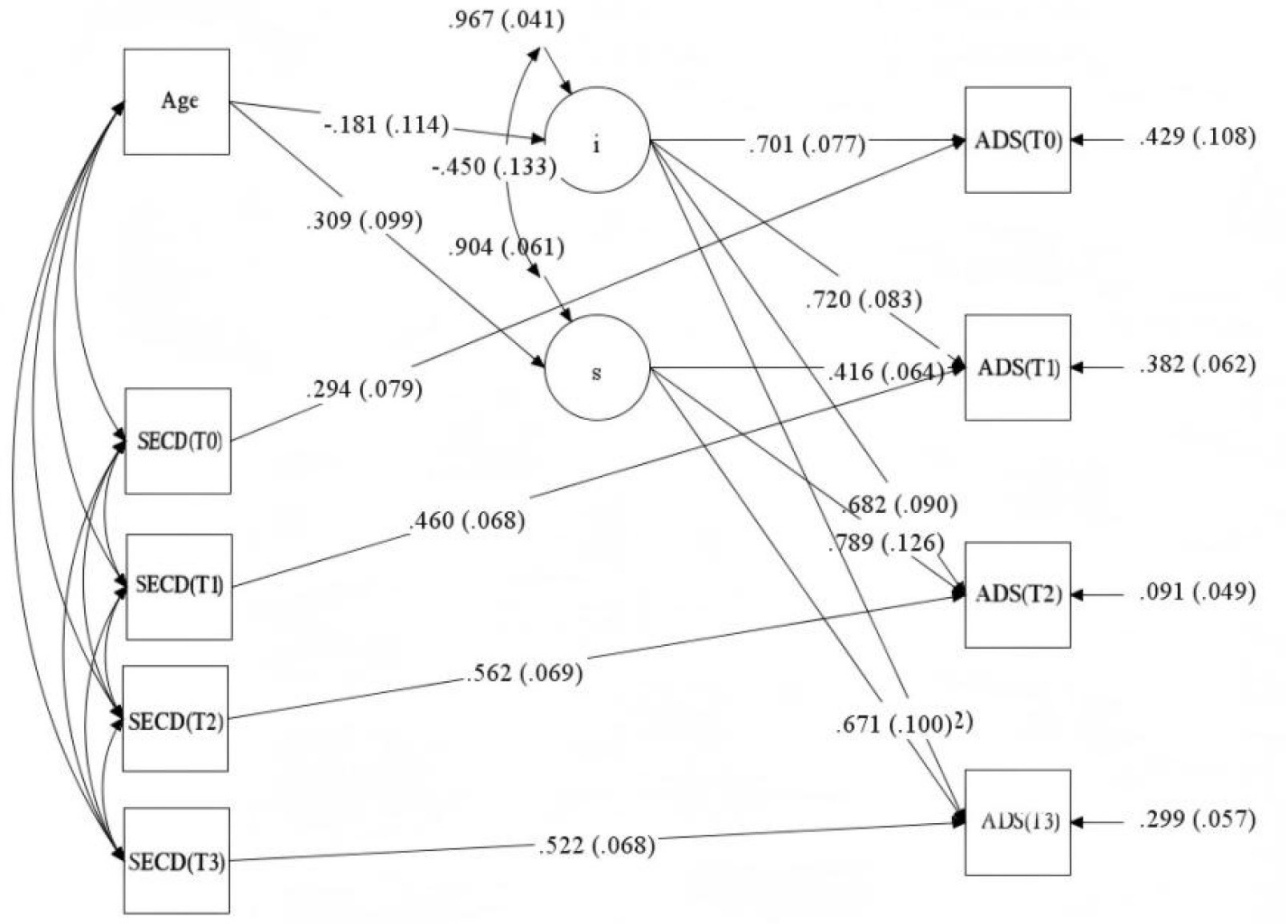
Path diagram of the full conditional latent growth model with standardized estimates.

The standardized parameter estimates (β) for the significant paths in the final full model are summarized in Table 4 and illustrated in Figure 2. Regarding time-varying effects, self-efficacy at each time point showed a strong, positive concurrent association with disability acceptance (β_(T0) = 0.294, *p* < 0.001; β_(T1) = 0.460, *p* < 0.001; β_(T2) = 0.562, *p* < 0.001; β_(T3) = 0.522, *p* < 0.001). Concerning time-invariant effects, age was a significant positive covariates of the slope factor (β = 0.309, *p* < 0.01), indicating that older patients experienced a steeper rate of increase in acceptance over time. The path from age to the intercept was negative but non-significant (β = −0.181, *p* > 0.05). In this final model, the covariance between the intercept and slope factors became significantly negative (β = −0.450, *p* < 0.001).

**Table 4:** Standardized regression coefficients (β) for the conditional latent growth models.

Covariates → outcome	Time-varying covariate model (β)	Time-invariant covariate model (β)	Full model (mixed covariates) (β)
Time-varying covariate (self-efficacy)			
SECD(T0) → ADS (T0)	0.284^***^	–	0.294^***^
SECD (T1) → ADS (T1)	0.463^***^	–	0.460^***^
SECD(T2) → ADS (T2)	0.577^***^	–	0.562^***^
SECD(T3) → ADS (T3)	0.535^***^	–	0.522^***^
Time-invariant covariate (age)			
Age → intercept (I)	–	−0.136	−0.181
Age → slope (S)	–	0.334^**^	0.309^**^
Covariance of latent variables			
Intercept (I) ↔ slope (S)	−0.477^***^	−0.181	−0.450^***^

T0 = discharge; T1 = 1 month; T2 = 3 months; T3 = 6 months. ADS = Acceptance of Disability Score. SECD = Self-Efficacy for Managing Chronic Disease—indicates the covariate was not included in that model.

^*^p < 0.05,

^**^p < 0.01,

^***^p < 0.001.

### 3.5 Supplementary analysis: baseline NIHSS

To assess whether initial stroke severity influenced the trajectory of disability acceptance, we added baseline NIHSS score as a time-invariant covariate in the full conditional latent growth model (see Supplementary Table S2). Results showed that baseline NIHSS was not significantly associated with either the initial level of disability acceptance (β = −0.106, *p* = 0.332) or its rate of change over time (β = 0.175, *p* = 0.116). The inclusion of NIHSS did not alter the magnitude or significance of the primary findings (self-efficacy concurrent associations and age effect on slope).

## 4 Discussion

This study employed latent growth modeling to longitudinally examine the developmental trajectory of disability acceptance over 6 months post-discharge in patients with Hypertensive Intracerebral Hemorrhage (HICH), and to investigate the effects of time-varying self-efficacy and time-invariant age on this trajectory. The key findings indicate that disability acceptance follows a nonlinear pattern of initial growth followed by stabilization; Self-efficacy showed a strong, time-specific positive association with concurrent disability acceptance; and while age did not significantly predict the initial level of acceptance, it positively predicted the rate of growth in acceptance over time.

### 4.1 Trajectory of disability acceptance

The study found that disability acceptance increased linearly during the first 3 months post-discharge (T0–T2), before reaching a plateau between the third and 6 months (T2–T3). This “growth-plateau” pattern aligns with findings on psychological adaptation in some chronic and neurological conditions. For instance, research on stroke recovery suggests that psychological adjustment and quality of life often show rapid improvement within the first 6 months post-event, after which the rate of gain typically slows or stabilizes (17). Our findings refine the understanding of this process specifically for the HICH population, indicating that disability acceptance, as an active psychological adjustment process, undergoes its most positive changes primarily in the early post-discharge period. This highlights a potential “critical window” for intervention within the first 3 months, during which targeted psychosocial support may be most beneficial.

### 4.2 Predictive role of self-efficacy

A central finding was that self-efficacy at each time point showed a strong, positive concurrent association with disability acceptance, after accounting for other factors. This result strongly supports Social Cognitive Theory in chronic disease management, which posits self-efficacy as a core mechanism influencing behavioral, cognitive, and emotional adaptation (18). Studies in stroke populations corroborate that self-efficacy is a key psychological resource facilitating coping, rehabilitation engagement, and quality of life (15, 19). By modeling self-efficacy as a time-varying covariate, our longitudinal design provides more precise evidence for its proximal and synchronous influence on acceptance. This suggests that enhancing a patient's confidence in managing symptoms, performing daily tasks, and coping emotionally at specific rehabilitation stages (e.g., at discharge, 1 month post-discharge) may be concurrently linked to greater acceptance of their disability (20).

### 4.3 Predictive role of age

An intriguing finding was that age did not significantly predict the initial level of acceptance but showed a significant positive association with the growth slope. This suggests that while younger and older patients may have similar levels of acceptance at discharge, older patients experience a faster rate of increase in acceptance over the subsequent months. This contrasts with perspectives suggesting older adults face greater psychological challenges due to resource loss and reduced adaptive capacity (21). Potential explanations include: older adults may draw upon richer life experiences and coping strategies (e.g., cognitive reappraisal), or may have more realistic expectations regarding age-related health changes, enabling quicker adjustment of mindset and goals when facing disability (22). Furthermore, Socioemotional Selectivity Theory suggests that with advancing age, motivational focus shifts toward emotion regulation and deriving meaning, which may facilitate quicker acceptance of unchangeable circumstances and a search for positive experiences (23). However, this finding requires replication in diverse cultural and clinical samples.

### 4.4 Implications for research and clinical practice

This study has important theoretical and practical implications. Theoretically, using LGMs, we validated a nonlinear, dynamic pattern of disability acceptance post-HICH and clarified the distinct roles played by self-efficacy (a state-like covariate) and age (a trait-like covariate). Practically, the findings inform the development of stage-specific and individualized psychological interventions. First, interventions targeting self-efficacy should be integrated throughout rehabilitation, with an emphasis on immediacy. Techniques such as goal-setting, skills modeling, review of mastery experiences, and positive feedback can be employed to boost confidence in various situations (24). In addition to traditional self-management education, recent meta-analytic evidence confirms that comprehensive digital health interventions can effectively improve self-management behaviors and clinical outcomes in patients with chronic conditions (25). Concurrently, support delivered by non-professional personnel, such as community health workers, has been established as an effective strategy for improving outcomes like self-efficacy Among Community-Dwelling Older Adults (26). This suggests that for HICH patients, integrating diversified support modalities may enhance the engagement and effectiveness of rehabilitation interventions. Second, considering age differences, younger patients might require more extended and in-depth psychological support to address grief over disrupted future life plans, whereas interventions for older patients could focus on strengthening their existing adaptive strategies and assisting them in finding new meaning post-disability (27).

### 4.5 Interpretation of the non-significant effect of baseline NIHSS

Contrary to our expectation, baseline stroke severity (NIHSS) was not significantly associated with either the initial level or the rate of change of disability acceptance. One possible explanation is that dynamic psychological factors, such as self-efficacy, exert a more proximal and powerful influence on adaptation than static clinical indicators measured at a single time point (18). This aligns with Social Cognitive Theory, which posits that perceived capabilities often outweigh objective severity in shaping behavioral and emotional outcomes. In stroke populations, self-efficacy has been shown to be a stronger covariate of psychological adaptation (e.g., quality of life, depression) than neurological impairment severity (17). Alternatively, the restricted range of NIHSS scores in our sample may have limited our ability to detect an effect, as patients with extremely severe deficits were excluded due to communication or cognitive impairments. In such a truncated range, even a true association may appear non-significant (28). Thus, the null finding does not rule out a potential influence of severe stroke on psychological adaptation, but suggests that within mild-to-moderate severity ranges, baseline clinical measures may be less predictive than ongoing psychological processes.

### 4.6 Limitations and future directions

First, the single-center design and modest sample size limit generalizability. We also lacked detailed data on post-discharge rehabilitation. Second, unmeasured confounders (e.g., social support, depression) and the observational design preclude causal inference. Third, NIHSS was measured only at admission, not during follow-up, so we could not track neurological recovery. Fourth, follow-up assessments were partly conducted via telephone, which may introduce social desirability or recall bias; nevertheless, the use of validated scales with good reliability partially mitigates this issue.

Future multicenter studies should include broader inclusion criteria, systematic tracking of rehabilitation, serial NIHSS assessments, and more frequent (e.g., in-person or diary-based) assessments to disentangle the temporal direction between self-efficacy and disability acceptance.

## 5 Conclusion

In conclusion, this study demonstrates that disability acceptance in HICH patients follows a nonlinear trajectory of initial growth and subsequent stabilization over 6 months post-discharge. Self-efficacy is a key proximal correlate of acceptance at each stage, while age influences the pace of change. These findings underscore the importance of implementing individualized psychological interventions, particularly during the critical early post-discharge period (first 3 months), that focus on enhancing self-efficacy and consider age-related differences, to optimize long-term psychological adaptation and overall rehabilitation outcomes.

## Data Availability

The original contributions presented in the study are included in the article/Supplementary material, further inquiries can be directed to the corresponding author.
